# Hepsin promotes breast tumor growth signaling via the TGFβ‐EGFR axis

**DOI:** 10.1002/1878-0261.13545

**Published:** 2023-11-13

**Authors:** Denis Belitškin, Pauliina Munne, Shishir M. Pant, Johanna M. Anttila, Ilida Suleymanova, Kati Belitškina, Daniel Kirchhofer, James Janetka, Taivo Käsper, Sami Jalil, Jeroen Pouwels, Topi A. Tervonen, Juha Klefström

**Affiliations:** ^1^ Research Programs Unit/Translational Cancer Medicine Research Program and Medicum, Faculty of Medicine University of Helsinki Finland; ^2^ Pathology Department North Estonia Medical Centre Tallinn Estonia; ^3^ Department of Early Discovery Biochemistry Genentech, Inc. South San Francisco CA USA; ^4^ Department of Biochemistry and Molecular Biophysics Washington University School of Medicine St. Louis MO USA; ^5^ Sixfold OÜ Tartu Estonia; ^6^ Stem Cells and Metabolism Research Program, Faculty of Medicine University of Helsinki Finland; ^7^ Foundation for the Finnish Cancer Institute, Helsinki & FICAN South Helsinki University Hospital Finland

**Keywords:** breast cancer, EGFR, *HPN*, mouse cancer model, patient‐derived explant culture model, TGFβ

## Abstract

Hepsin, a type II transmembrane serine protease, is commonly overexpressed in prostate and breast cancer. The hepsin protein is stabilized by the Ras‐MAPK pathway, and, downstream, this protease regulates the degradation of extracellular matrix components and activates growth factor pathways, such as the hepatocyte growth factor (HGF) and transforming growth factor beta (TGFβ) pathway. However, how exactly active hepsin promotes cell proliferation machinery to sustain tumor growth is not fully understood. Here, we show that genetic deletion of the gene encoding hepsin (*Hpn*) in a WAP‐Myc model of aggressive MYC‐driven breast cancer inhibits tumor growth in the primary syngrafted sites and the growth of disseminated tumors in the lungs. The suppression of tumor growth upon loss of hepsin was accompanied by downregulation of TGFβ and EGFR signaling together with a reduction in epidermal growth factor receptor (EGFR) protein levels. We further demonstrate in 3D cultures of patient‐derived breast cancer explants that both basal TGFβ signaling and EGFR protein expression are inhibited by neutralizing antibodies or small‐molecule inhibitors of hepsin. The study demonstrates a role for hepsin as a regulator of cell proliferation and tumor growth through TGFβ and EGFR pathways, warranting consideration of hepsin as a potential indirect upstream target for therapeutic inhibition of TGFβ and EGFR pathways in cancer.

## Graphical abstract

Serine protease hepsin, which is commonly overexpressed in prostate, ovarian, and breast cancers, promotes cancer progression with yet unclear mechanisms. We show with breast cancer patient‐derived explant cultures (PDECs), Myc‐driven mouse mammary tumors, and cell culture models that ablation of hepsin activity inhibits tumor growth and cell proliferation through loss of TGFβ and EGFR signaling in breast cancer context.

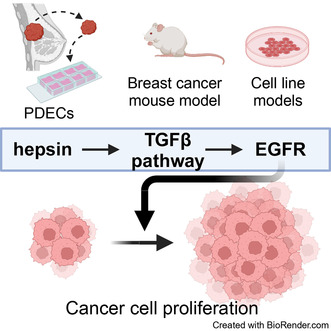

**Abbreviations**
BRB‐seq: Bulk RNA barcoding and sequencing
cDNA: complementary DNA
DAB: 3,3′‐diaminobenzidine
Dox: doxycycline
ECM: extracellular matrix
EGFR: epithelial growth factor receptor
GSEA: gene signature enrichment analysis
HGF: hepatocyte growth factor
IHC: immunohistochemical staining
KO: knockout
MAPK: mitogen‐activated protein kinase
MCF10A‐HT cells: MCF10A‐pIND20‐HPN pL6‐TGFβ1V5 cells
MCF10A‐pIND20‐HPN cells: MCF10A cells engineered to express doxycycline (dox)‐inducible hepsin
MSP: macrophage stimulating protein
ORF: open reading frame
PDEC: patient‐derived explant culture
PDGF: platelet‐derived growth factor
TGFβ: transforming growth factor beta
TTSP: type II transmembrane serine protease
WT: wild‐type

## 1 Introduction

Hepsin, a member of the Type II Transmembrane Serine Proteases (TTSPs) family, is frequently overexpressed in the prostate [1], ovarian [2], and breast cancer [3, 4]. Functions of this protease include regulation of epithelial integrity [3, 4, 5] and proteolytic activation of hepatocyte growth factor (HGF) and macrophage stimulating protein (MSP) [6, 7]. We recently demonstrated that hepsin regulates transforming growth factor β (TGFβ) signaling in the murine mammary gland and breast cancer models through a release of latent TGFβ from extracellular matrix (ECM) storage [8]. In models of breast cancer, hepsin is required for oncogenic Ras‐mediated loss of epithelial integrity and tumor progression [4]. Furthermore, in prostate cancer models, hepsin overexpression promotes metastatic dissemination without notably affecting primary tumor growth [1, 9]. Moreover, in WAP‐Myc model of aggressive MYC‐driven breast cancer, an inducible hepsin overexpression decreases tumor latency concomitantly with loss of epithelial integrity [3]. Taken together, the current data suggest a critical role for hepsin in mediating tumor progression but it is unclear whether hepsin acts primarily to invasive and metastatic processes through breakdown or modulation of the extracellular matrix or whether the overexpression of hepsin also directly promotes tumor cell proliferation through activation of growth‐promoting signaling pathways. New insight into these open questions would allow more definitive assessment of the potential of hepsin as a target for anticancer drug development.

Here, we explored the role of hepsin in cancer cell growth signaling using a syngrafted mouse model of hepsin‐deficient breast cancer, genetically engineered breast cancer cell lines, and patient‐derived breast cancer explant cultures (PDECs) exposed to specific inhibitors of hepsin. We report that hepsin promotes cell proliferation by activating the TGFβ epidermal growth factor receptor (TGFβ‐EGFR) signaling axis. In a WAP‐Myc mouse model of breast cancer, hepsin knockout reduced mammary tumor growth at the primary and metastatic sites, accompanied by downregulation of TGFβ, EGFR, and mitogen‐activated protein kinase (MAPK) signaling, whereas in the mammary tumors with inducible hepsin overexpression the same signaling pathways were upregulated. We also demonstrate that hepsin‐induced cell proliferation is coupled to TGFβ‐EGFR signaling in the 3D cultures of mammary epithelial cells. Finally, we demonstrate that a hepsin function‐blocking antibody Ab25 [10] and a hepsin small molecule inhibitor ZFH7116 [11, 12] can be used as molecular tools to downregulate TGFβ signaling and EGFR protein levels in PDECs. Thus, the type II transmembrane protease hepsin may offer an indirect yet highly accessible extracellular route to inhibit TGFβ/EGFR pathway in cancer.

## 2 Materials and methods

### 2.1 Cell lines

Cell lines were regularly tested for mycoplasma contamination and authenticated by using GenePrint 24 System kit (Promega, Cat #B1870, Madison, WI, USA) at Institute for Molecular Medicine Finland (FIMM) Genomics unit (HiLIFE infrastructures, University of Helsinki and Biocenter Finland). The MCF10A (ATCC Cat# CRL‐10317, RRID:CVCL_0598) cell line was obtained from the American Type Culture Collection and cultured as described previously [3] in MCDB 170 (US Biological, Salem, MA, USA) supplemented with 5 μg·mL^−1^ insulin, 70 μg·mL^−1^ bovine pituitary extract, 0.5 μL·mL^−1^ hydrocortisone, 5 ng·mL^−1^ EGF, 5 μg·mL^−1^ human transferrin, and 0.01 μm isopropretenol (all from Sigma Aldrich, St.Louis, MO, USA). The MCF10A‐based cell lines harboring the pLenti6‐TGFβ1V5 and inducible hepsin overexpression constructs were published previously [3, 8].

### 2.2 RNASeq from mouse and human tumor tissue samples

RNA was extracted from tumors using Precellys hard‐tissue beads and TRIzol. RNA was then DNase treated and purified using RNeasy Mini Kit from Qiagen (Germantown, MD, USA).

The Bulk RNA barcoding and sequencing (BRB‐seq) was performed as described before [13]. RNA sample (10 ng) barcoding was performed with Indexing Oligonucleotides (Integrated DNA Technologies, Coralville, IA, USA). Complementary DNA (cDNA) was prepared with RT mix containing Maxima RT buffer, 1 mm dNTPs, Maxima H‐RTase (all ThermoFisher Scientific, Waltham, MA, USA), and Template Switch Oligo (Integrated DNA Technologies). RNAse inhibitor used was RiboLock (ThermoFisher Scientific).

cDNA amplification was done with PCR using RT mix as a template, SMART PCR primer, 1× HiFi, HotStart Readymix (Kapa Biosystems, Wilmington, MA, USA). The samples were thermocycled in a T100 thermocycler (BioRad, Hercules, CA, USA). According to the manufacturer's instructions, the PCR products were pooled together in sets of 12 samples containing different Indexing Oligos and purified with 0.6× Agencourt AMPure XP beads (Beckman Coulter, Brea, CA, USA). The purified cDNA was fragmented using the Nextera kit. The reaction was performed according to the manufacturer's instructions, apart from the P5 SMART primer instead of the S5xx Nextera primer.

Qubit 2 fluorometer (Invitrogen, Carlsbad, CA, USA) was used for concentration measurements with the Qubit DNA HS Assay Kit (ThermoFisher Scientific). Library quality estimates were obtained using the LabChip GXII Touch HT (PerkinElmer, Shelton, CT, USA), with the DNA High Sensitivity Assay (PerkinElmer) and the DNA 5 K/RNA/Charge Variant Assay LabChip (PerkinElmer). Sequencing was performed with Illumina NextSeq 500, with a custom primer producing read 1 of 20 bp and read 2 (paired‐end) of 50 bp (Sequencing was performed at the Functional Genomics Unit of the University of Helsinki, Finland).

Oligonucleotide sequences: SMART PCR primer: AAGCAGTGGTATCAACGCAGAGT P5 SMART primer: AATGATACGGCGACCACCGAGATCTACACGCCTGTCCGCGGAAGCAGTGGTATCAACGCAGAGT*A*C TSO: AAGCAGTGGTATCAACGCAGAGTGAATrGrGrG Sequencing read 1: GCCTGTCCGCGGAAGCAGTGGTATCAACGCAGAGTAC.

Analysis of the data was performed using gsea 4.1.0 [14]. Clustering of the gene sets was analyzed and illustrated using cytoscape [15].

### 2.3 qRT‐PCR

After the RNA isolation from tumors (described above), cDNA synthesis was performed using iScript™ Reverse Transcription Supermix following manufacturer's instructions (Bio‐Rad, #1708840). Primer sequences for mouse *Egfr* were obtained from PrimerBank (ID: 10880776a1). DNA oligo primers were purchased from Sigma‐Aldrich/Merck (Darmstadt, Germany). For measuring *Egfr* mRNA levels, the qPCR was performed with iQ™ SYBR Green Supermix according to instructions of the manufacturer (Bio‐Rad, #1708882) in a CFX Duet Real‐Time PCR System (Bio‐Rad). The reaction conditions were as follows – initial denaturation: 95 °C, 3 min and denaturation and annealing (40 cycles): 95 °C, 10 s; 61 °C, 30 s, 72 °C (single), 10 s. Relative mRNA levels of *Egfr* were obtained by comparing PCR cycles to *Gapdh* using the ΔΔ*C*_T_ method and normalizing the samples to control genotype. Negative controls (H_2_O and negative reverse transcriptase control) were included in the assay and all samples were tested in three technical replicates.

### 2.4 3D cell and PDEC culture experiments

MCF10A growth experiments were performed in Cultrex RGF Basement Membrane Extract, Type 2 (R&D Systems, 3533‐005‐02, Minneapolis, MN, USA) 3D matrix and serum‐free media as described in [3, 8]. Human tumor samples were obtained from elective breast cancer surgeries between May 2019 and May 2020 at the Helsinki University Central Hospital (Ethical permit: 243/13/03/02/2013/TMK02 157 and HUS/2697/2019 approved by the Helsinki University Hospital Ethical Committee). The procedure for Patient Derived Explant Cultures (PDECs) starts from primary breast tumors that come fresh from the elective breast cancer surgeries. Isolated tumor samples are incubated overnight in collagenase A (1 mg·mL^−1^; Sigma) containing the MammoCult media (StemCell technologies, Vancouver, Canada) with gentle shaking (130 r.p.m.) at +37 °C. The resulting explants are collected via centrifugation at 353 rcf for 3 min and washed once with the culture medium. The size of the tumor fragments varies in size (diameter between 20 and 250 μm). The procedures for preparing explant cultures have been described in detail elsewhere [16, 17, 18]. In brief, 10 μL of explants was mixed with 40 μL per well of Cultrex on an 8‐well chamber slide. 0.5 mL of Mammocult media was added per 8‐chamber slide well after the matrix was solidified. The 0.1% DMSO was used as a control, and 5 μm Ab25 was added to the cultures for 48 h.

The patients participated to this study by signing a written consent form designed according to Declaration of Helsinki and the study methodologies conformed to the standards set by the Declaration of Helsinki.

### 2.5 Antibodies and inhibitors

The inhibitors used in this study were K02288 (Selleckchem, S7359, Houston, TX, USA), Galunisertib/LY2157299 (Selleckchem, S2230), RepSox (Sigma‐Aldrich/Merck, R0158), A‐83‐01 (MedChem Express, HY‐10432, Monmouth Junction, NJ, USA), Erlotinib (EGFRi, Selleckchem, S1023), and human ALK1 inhibitory antibody (R&D Systems, MAB3701). The hepsin inhibitory antibody (Ab25) was a kind gift from Dr. Kirchhofer, Genentech [10]. ZFH7116 provided by Dr. Janetka [12]. The antibodies used in this study were Anti‐sheep HRP (Upstate cell signaling solutions, #12‐342), anti‐rabbit HRP (Millipore, AP132P, Burlington, MA, USA), anti‐hepsin (R&D Systems, AF4776), anti‐GAPDH (CST#2118S), anti‐β‐tubulin (Abcam, ab6046, Cambridge, UK), anti‐pEGFR Y1068 (Abcam, ab40815), anti‐EGFR (Abcam, ab32077), anti‐pSmad2/3 (CST#8828), anti‐Smad2/3 (CST#8685), anti‐Smad2 (CST#5339), anti‐pH3 (Santa Cruz, sc‐8656, Dallas, TX, USA), total‐H3 (CST#9715), anti‐V5 (Invitrogen, #R96025), anti‐Ki67 (Abcam, ab15580), anti‐cleaved caspase 3 (CST#9661), anti‐F‐actin (phalloidin staining) (Thermo Fisher Scientific, A22283), anti‐pSmad1/5 (CST#9516) and anti‐ALK‐1 (R&D Systems, AF370).

### 2.6 Animal models and experiments

The FVB mice were originally obtained from Janvier‐Labs and all mice used for the experiments at the Laboratory Animal Center, HiLIFE, University of Helsinki and Biocenter Finland, were housed in individually ventilated cages under the optimal conditions of temperature and humidity. The experiments were conducted according to 3R principles, and animal welfare was regularly monitored.

The Experiments were approved by Experimental animals The National Animal Ethics Committee of Finland (ESAVI/3678/04.10.07/2016).

The *Hpn* KO strain (FVB‐*Hpn* ^em1JKle^) was generated in‐house by CRISPR‐CAS9 technique as described in [8]. For experiments, 8–10 weeks old WAP‐Myc mice (FVB.Cg‐Tg(WapMyc)212Bri/J from Jackson laboratory) were crossed with *Hpn* KO mice. The resulting WAP‐Myc; *Hpn* KO female mice were exposed to two pregnancies to activate WAP promoter, and consequently Myc driven tumor growth. The tumor latency for WAP‐Myc;*Hpn* KO tumors was approximately similar to WAP‐Myc WT tumors (about 6‐months from the birth with two pregnancies). The syngrafting experiments were performed essentially as described in [19]. In short, tumor cells were isolated from single female mice carrying WAP‐Myc WT or WAP‐Myc;*Hpn* KO tumors and stored frozen in –140 °C. To generate tumor syngrafted cohorts, cells derived from the tumors were thawed day before transplantation as floating culture and their viability was controlled by Trypan Blue before transplantation. The tumor cells were syngrafted orthotopically as single cells to the cleared mammary fat pads of virgin 5‐week‐old female FVB WT recipient mice; 0.1 million cells per each gland. Cells were transplanted bilaterally to the glands number 4 and 5 of each recipient mice. Recipient mice did not undergo any pregnancies and the final mouse in the experiment was sacrificed less that hundred days after the initial transplantation. The mice with doxycycline‐inducible hepsin have been previously described in [3, 8]. In short, to generate WAP‐Myc syngraft tumors with doxycycline‐inducible hepsin, we lentivirally transduced pIND21‐HPN construct, which contains hepsin ORF under a doxycycline regulated promoter, into isolated WAP‐Myc mammary epithelial cells at MOI 10. To generate cohorts of mice, the transduced cells were injected into the cleared fat pads of 3‐week‐old female FVB recipient mice (100 000 transduced cells per gland). To activate hepsin overexpression 3 days after the transplantation, the mice started to receive doxycycline in their drinking water (also containing 5% sucrose) and the control mice received sucrose water only. WAP‐Myc was activated and tumorigenesis initiated at week 8 by two sequential pregnancies in the fat pad‐transplanted female mice. The mice were killed when the tumor diameter reached 1 cm.

*Ex vivo* treatment WAP‐Myc tumor cells was performed in growth media [20] without serum for 6 h with 0.1 or 20 ng·mL^−1^ TGFβ1.

### 2.7 Immunohistochemistry and image analysis

Tumor tissues were fixed with 4% paraformaldehyde and embedded in paraffin. The samples were cut into 5 μm slices and deparaffinized. The heat‐induced antigen retrieval was performed in the microwave for anti‐Ki67 and in the oven (+60 °C) for anti‐cleaved caspase 3. Antigen retrieval was performed with a citrate buffer solution (Dako). Histochemical stainings were carried out using standard techniques for IHC. Digital images from IHC‐stained sections were obtained with a Leica DM LB microscope with digital camera. The digital images were transported to ImageJ (2.0.0‐rc‐69/1.52n) where Color deconvolution tool was used to separate hematoxylin and 3,3′‐diaminobenzidine (DAB) channels. These separate figures were then thresholded and Analyze particles tool was used to obtained the total area of hematoxylin positive cells and DAB (Ki67 or cleaved caspase 3) positive cells on each image of a tumor section.

### 2.8 Immunoblot analysis

Immunoblot analysis was performed as described previously [8]. In short, all lysates for immunoblot analysis were prepared by lysing cells in PBS‐1%TritonX‐100 buffer supplemented with Protease Inhibitor Cocktail and Phosphatase inhibitor (Roche, Basel, Switzerland). After that, the resulting suspension was incubated on ice for 10 min, centrifuged, and the supernatant collected. Tissue lysates were made in the mentioned lysis buffer using Precellys 24 tissue homogenizer (Bertin instruments) and the Precellys kit (#KT03961‐1‐003.2). Proteins were separated on gradient SDS gels (BioRad) and transferred to nitrocellulose membranes (BioRad #1704158). Transfer quality was evaluated with Ponceau staining (Sigma, #P7170). Immunoblots were quantitated using image j (Bethesda, MD, USA). Statistical significance was determined via a one‐sided t‐test, unless specified otherwise in the figure legends.

## 3 Results

### 3.1 Genetic deletion of *Hpn* reduces MYC‐driven mouse mammary tumor growth in the mammary fat pad and lung metastases

**Fig. 1:**
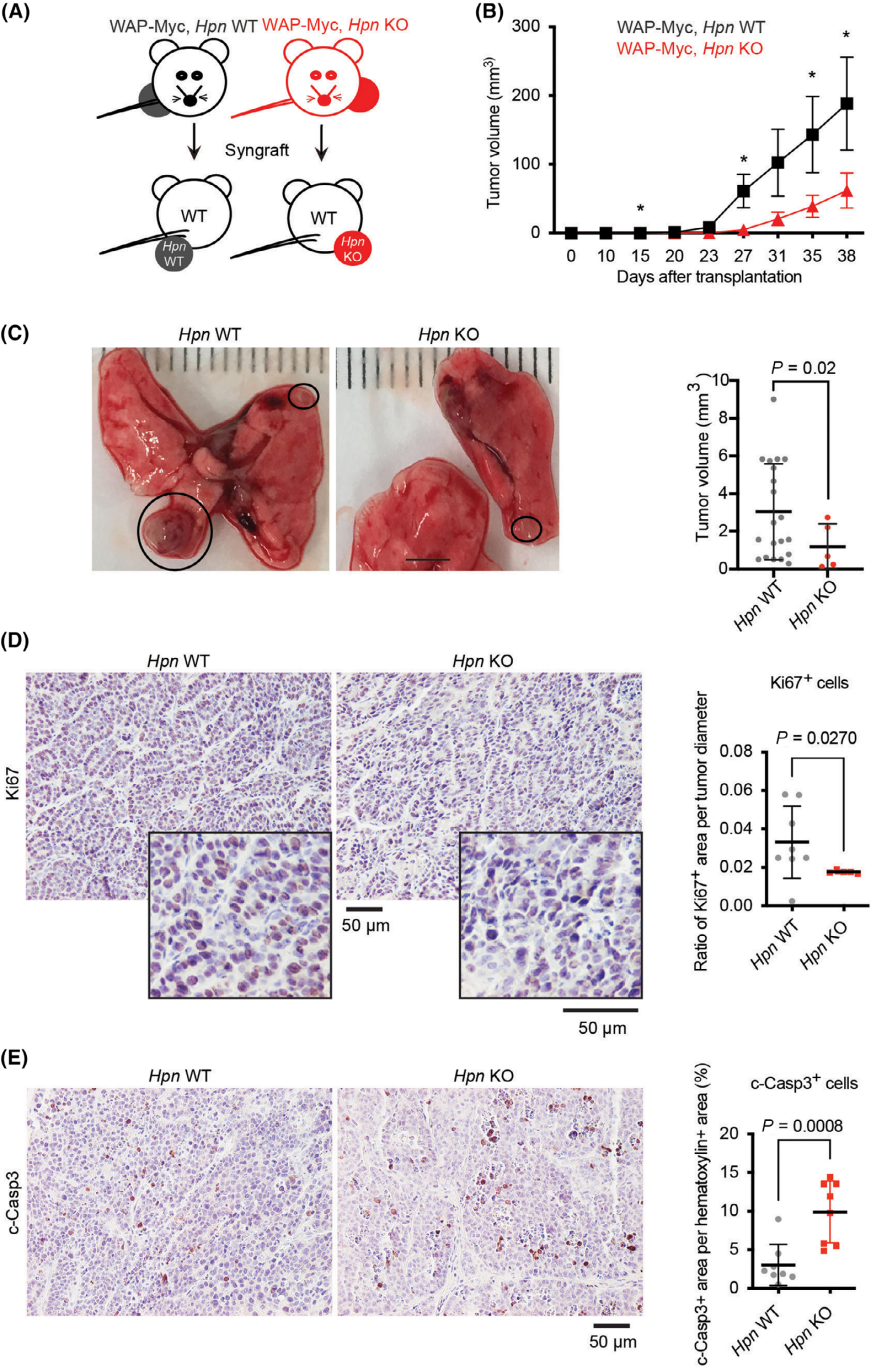
Loss of hepsin reduced primary and metastatic mouse mammary tumor growth. (A) Schematic representation of the orthotopic transplantation of wild‐type WAP‐Myc tumor cells (WAP‐Myc, *Hpn* WT; black) and hepsin knockout WAP‐Myc tumor cells (WAP‐Myc, *Hpn* KO; red) into WT recipients. In all experiments, both left and right flanks injected with tumor cells. (B) Growth curves of *Hpn* WT (*N* = 16) and *Hpn* KO (*N* = 14) WAP‐Myc tumors in *Hpn* WT recipient mice. Tumor volumes are presented as averages (mean ± SEM) per group, the timeline specifying days after the transplantation. Statistical significance from student's *t*‐test, * denotes *P* < 0.05. (C) Representative photographs of lungs from *Hpn* WT and *Hpn* KO groups with metastatic tumor nodules indicated by black circles. The scale bar is 3 mm. The graph (on the right) shows the volume of single metastatic lung tumors (mean ± SD, *N* = 20 for *Hpn* WT and *N* = 5 for *Hpn* KO). One outlier from each group was removed following the Iglewicz and Hoaglin's robust test for multiple outliers (two‐sided test; Outlier criterion: Modified Z score ≥ 3.5). Statistical significance analyzed by a student's *t*‐test. The raw data without exclusion of the outliers is shown in Fig. S1D. (D) Representative images with magnified inserts of *Hpn* WT and *Hpn* KO WAP‐Myc FFPE tumor sections with IHC staining (brown) for cleaved caspase 3 (c‐Casp3). The graph on the right, shows quantitation of Ki67^+^ cell area as proportion of total cell area (nuclei) in the sections (hematoxylin counter staining, WT *N* = 9 and KO *N* = 8, mean ± SEM). The graph shows the proportion of Ki67^+^ cell area to whole tumor diameter (mm) when the mice were sacrificed (WT *N* = 8 and KO *N* = 5, mean ± SEM). (E) Representative images of *Hpn* WT (*N* = 8) and *Hpn* KO WAP‐Myc (*N* = 8) FFPE tumor sections with IHC staining (brown) for cleaved caspase 3 (c‐Casp3). The graph shows quantitation of c‐Casp3^+^ area as a proportion of total cell area (nuclei) in the sections (mean ± SEM, field of view, hematoxylin counter staining). One outlier in (D) and one outlier in (E) were removed by using a ROUT (Q = 1%) test and the statistical significance was tested with Welch's corrected unpaired *t*‐test. ns, not significant.

To determine the role of hepsin in mouse mammary tumorigenesis and metastasis, we used the WAP‐Myc syngeneic mouse tumor model [3, 17, 19, 21]. We syngrafted WAP‐Myc mammary tumor cells with *Hpn* wild‐type (*Hpn* WT) or *Hpn* knockout (*Hpn* KO) background into mammary fat pads of WT recipient mice (Fig. 1A). The overall survival of the *Hpn* KO tumor bearing mice was not significantly altered (Fig. S1A), however, the primary *Hpn* KO mammary tumors displayed significantly reduced growth for about one month after the transplantation and a trend for slower growth over the 2 months follow‐up period (Fig. 1B and Fig. S1B). While the endogenous WAP‐Myc tumors rarely metastasize [20], the syngrafted WAP‐Myc tumors can metastasize to the lungs [19]. In our experiments, most of the syngrafted mice developed metastatic nodules in the lungs at the time of sacrifice. The frequency of metastasis was similar for both the *Hpn* WT and the *Hpn* KO group; 6 out of 9 syngrafted mice formed metastasis. We did observe a trend towards a reduced incidence of metastases in the *Hpn* KO, although the results were not powered by statistical analyses (Fig. S1C). However, the *Hpn* KO metastatic tumors were clearly smaller in size when compared to WT ones (Fig. 1C and Fig. S1D). Next, we investigated the basic mechanisms behind the decreased size of Hpn KO tumors. We assessed cell proliferation by immunohistochemical (IHC) staining with Ki67 in primary tumor sections and found that the KO sections had significantly less Ki67 positive cells per tumor diameter (Fig. 1D). To investigate cell death, we did IHC staining with cleaved caspase 3 antibody in tumor sections and discovered that there is an increased staining for this apoptosis marker in KO sections (Fig. 1E). Furthermore, we observed by western blot analysis that there was a trend towards increased level of cleaved caspase 3 protein and hematoxylin and eosin staining pointed towards increased necrosis in the KO tumors as well (Fig. S1E,F). Therefore, both decreased proliferation and increased cell death possibly together contribute to reduced size of *Hpn* KO tumors in this mouse tumor model. Together, these data demonstrate that hepsin is necessary for a full potential of MYC to drive tumor growth in the primary site of transplantation (fat pad) and the growth of the metastases in the lungs.

### 3.2 Hepsin promotes MEK, EGFR, and TGFβ pathway signaling in WAP‐Myc mammary tumors

**Fig. 2:**
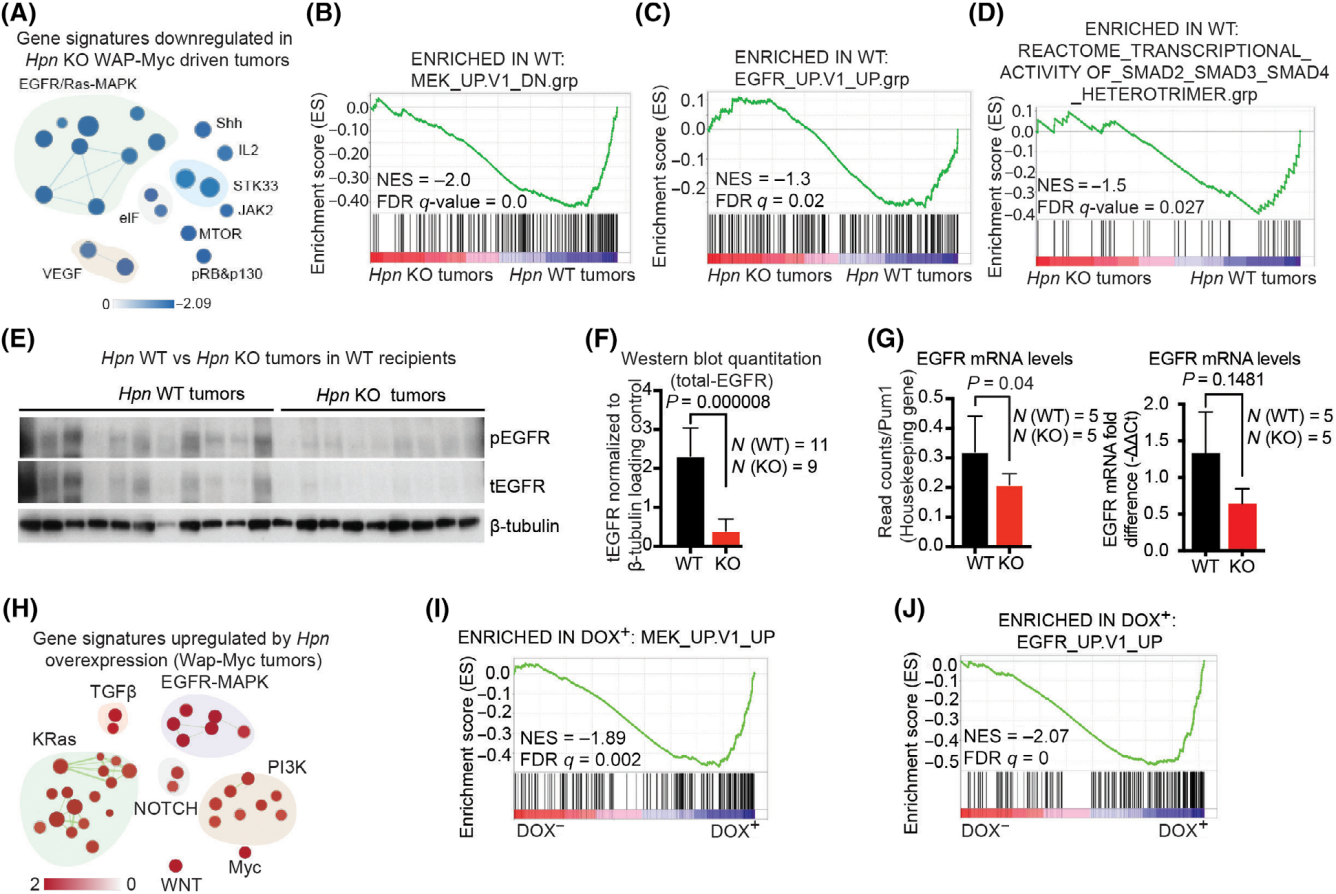
Hepsin regulates TGFβ, MAPK, and EGFR pathway signaling in the WAP‐Myc tumor model. (A) Selected gene signatures downregulated by loss of hepsin in WAP‐Myc driven tumors. The size of the nodes indicates the number of genes within a signature. The blue  color indicates the downregulation induced by hepsin knockout. (B–D) Gene set enrichment analysis (GSEA) corresponding to MEK_UP.V1_DN (MAPK kinase), EGFR_UP.V1_UP (Epidermal Growth Factor receptor) and REACTOME_TRANSCRIPTIONAL_ACTIVITY OF_SMAD2_SMAD3_SMAD4_HETEROTRIMER.grp (Transforming growth factor beta signaling) molecular signatures (NES‐normalized enrichment score, FDRq‐false discovery rate corrected significance). (E) Western blot analysis of *Hpn* WT (*N* = 11 tumors) and *Hpn* KO (*N* = 9 tumors) WAP‐Myc mammary tumor lysates for phosphorylated‐EGFR (pEGFR, pY1068) and total‐EGFR (tEGFR) with β‐tubulin as a loading control. Each lane represents one tumor. (F) Graph showing quantification of the western blot shown in (E). Total‐EGFR band density normalized to the β‐tubulin loading control represented as mean ± SD. Statistical significance was tested using the student's *t*‐test. (G) RNAseq‐derived EGFR mRNA levels (on the left), normalized to housekeeping gene Pum1, and qRT‐PCR‐derived normalized EGFR mRNA levels (on the right) in *Hpn* WT (*N* = 5 tumors) and *Hpn* KO (*N* = 5 tumors) groups. Data are presented as mean ± SD on the left and, on the right, as mean ± SEM. Statistical significance was tested using the student's *t*‐test and Welch's corrected unpaired *t*‐test. (H–J) GSEA of public data (published in ref. [8]; GEO dataset GSE164510), comparing mammary tumors overexpressing hepsin (*N* = 5; DOX^+^) to control tumors (*N* = 5; DOX^−^). The size of the nodes indicates the number of genes within a node. The red color indicates the upregulation induced by hepsin overexpression. (I, J) GSEAs corresponding to MEK_UP.V1_UP and EGFR_UP.V1_UP molecular signatures (details in H–J above).

To identify molecular pathways that could explain the reduced growth of the hepsin knockout tumors, we analyzed the transcriptomes of *Hpn* WT and *Hpn* KO primary WAP‐Myc tumors (Fig. 1B). Gene Signature Enrichment Analysis (GSEA) revealed specific downregulation of several cell growth pathway associated signatures in *Hpn* KO tumors, most notably the EGFR and MEK (MAPKK) pathways (Fig. 2A–C and Fig. S2A). Consistent with our previous findings [8], we also observed downregulation of TGFβ signaling in *Hpn* KO tumors (Fig. 2D). In immunoblot analysis, both phosphorylated‐EGFR (pY1068) and total‐EGFR levels were reduced in *Hpn* KO tumors (Fig. 2E,F). Furthermore, also the EGFR mRNA levels were downregulated in *Hpn* KO tumors as compared to *Hpn* WT tumors (Fig. 2G), suggesting a transcriptional component in EGFR downmodulation. We next asked if an ectopic induction of hepsin could promote the activity of MAPK, EGFR, and TGFβ pathways. To address the question, we analyzed transcriptomic data corresponding to WAP‐Myc transgene driven mammary tumors with or without doxycycline‐induced hepsin ([8]; GEO dataset GSE164510). We previously showed that overexpression of hepsin in WAP‐Myc transgene mouse model upregulated TGFβ pathway [8]. Strikingly, induction of hepsin in WAP‐Myc tumors led to a clear enrichment of the gene expression signatures corresponding to MAPK, EGFR, and in addition to previously observed TGFβ pathway signaling (Fig. 2H–J and Fig. S2B). Taken together, these data demonstrate that hepsin strongly regulates TGFβ, MAPK, and EGFR signaling pathways in context of MYC‐driven mammary tumorigenesis.

### 3.3 Hepsin regulates EGFR‐MAPK signaling and proliferation in a TGFβ dependent manner

**Fig. 3:**
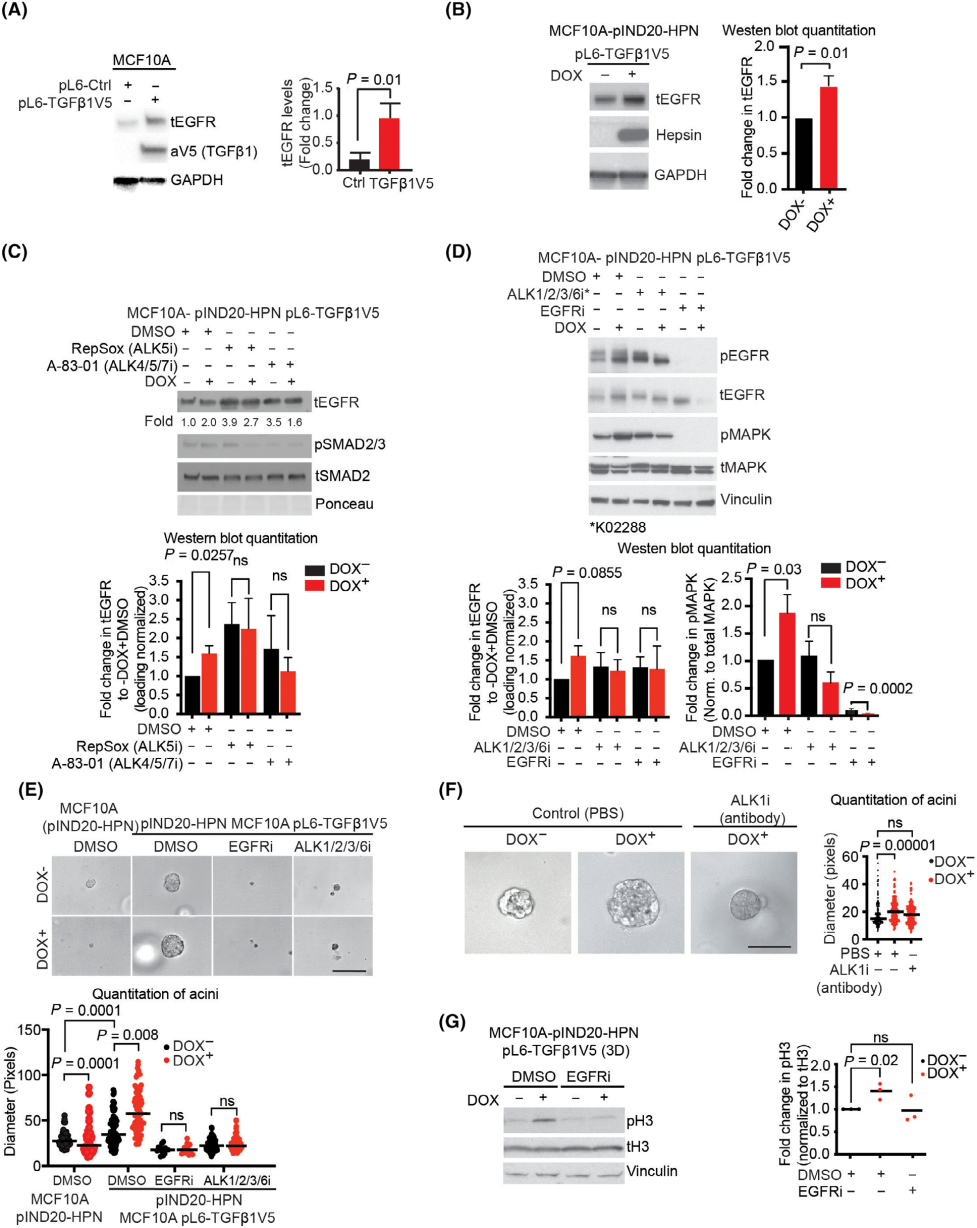
Hepsin regulates EGFR signaling in a TGFβ‐dependent manner. (A) Western blot comparison of total‐EGFR (tEGFR) levels in control MCF10A pL6‐Ctrl (*N* = 3) and MCF10A pL6‐TGFβ1V5, V5‐tagged TGFβ1 overexpressing cells (*N* = 3), which secrete around 80–100 pg·mL^−1^ of TGFβ1. Data are presented as mean ± SD. Significance was tested using the student's t‐test. (B) Western blot analysis of tEGFR in control doxycycline‐ (DOX^−^) (*N* = 3) and hepsin overexpressing (DOX^+^; *N* = 3) MCF10A‐ pIND20‐HPN pL6‐ TGFβ1V5 cells. Data are presented as mean ± SD. Significance was tested with the student's *t*‐test. (C) Western blot analysis of tEGFR, phospho‐SMAD2/3 (pSMAD2/3), and total‐SMAD2 (tSMAD2) in MCF10A‐pIND20‐HPN pL6‐TGFβ1V5 cells. The cells were treated with ALK5 inhibitor (i) (10 μm RepSox, *N* = 4) and ALK4/5/7 inhibitor (5 μm A‐83‐01, *N* = 3) or DMSO (control, *N* = 4) for 48 h with (DOX^+^; 1 μg·mL^−1^) or without (DOX^−^, control) hepsin overexpression (the numbers below the tEGFR blot indicate loading normalized values for tEGFR band intensity fold leftmost control lane). The histogram data are presented as mean ± SD. Significance was tested using unpaired *t*‐test. (D) Western blot analysis of phospho‐EGFR (pEGFR), tEGFR, total‐MAPK (tMAPK) and phospho‐MAPK (pMAPK), and vinculin (loading control) in MCF10A‐pIND20‐HPN pL6‐TGFβ1V5 cells. The cells were treated with the ALK1/2/3/6 inhibitor (i) (10 μm K02288), EGFR inhibitor (i) (10 μm Erlotinib), and DMSO (control) for 48 h. Data are presented as mean ± SD. Significance was tested with the student's *t*‐test (*N* = 3). (E) Phase‐contrast microscopy images of MCF10A‐pIND20‐HPN cells and MCF10A‐pIND20‐HPN pL6‐TGFβ1V5 (overexpressing TGFβ1) cells grown in 3D culture for 2 weeks with (DOX^+^; 1 μg·mL^−1^) or without (DOX^−^) induction of hepsin overexpression. Cells were treated with 10 μm EGFR or ALK1/2/3/6 inhibitor for 2 weeks as indicated in the figure. Scale bar represents 100 μm. (F) Phase‐contrast microscopy images of MCF10A‐pIND20‐HPN pL6‐TGFβ1V5 cells grown in 3D culture for 2 weeks in the presence of ALK1 inhibitory antibody (25 μg·mL^−1^). Scale bar represents 50 μm. For E and F, the experiments were repeated three times, with at least 100 epithelial structures counted per group in each repeat (one dot represents one structure), and black lines denote the mean values of each group. Significance was tested using the student's *t*‐test. (G) Western blot analysis of the mitotic marker phospho histone H3 (pH3 S10) MCF10A‐pIND20‐HPN pL6‐TGFβ1V5 cell line grown in 3D culture in the presence of the EGFRi (1 μm, 24 h) or DMSO. The quantification of blots of three independent experiments is shown in the graph (on the right), where the black lines denote the mean of each group. Significance was tested using the student's *t*‐test. ns, not significant.

Earlier studies have shown evidence that TGFβ pathway signaling promotes upregulation of total‐EGFR (tEGFR) in certain cancers, including breast cancer [22, 23, 24]. Furthermore, our earlier study reveals that hepsin regulates TGFβ signaling via fibronectin proteolysis [8]. Since the gene signatures for both TGFβ pathway and EGFR were enriched in WAP‐Myc tumors followed by induction of hepsin, and hepsin can activate TGFβ signaling, we asked if hepsin might use TGFβ pathway for activation of EGFR. Consistent with the earlier findings, we observed that engineered overexpression of TGFβ1 in mammary epithelial MCF10A cells (Fig. 3A, *P* < 0.05) or addition of recombinant TGFβ1 to WAP‐Myc cells (Fig. S3A, trend) enhanced the total EGFR (tEGFR) levels in these cells.

Next we asked if hepsin upregulates tEGFR levels in MCF10A cells engineered to express doxycycline (dox)‐inducible hepsin (MCF10A‐pIND20‐HPN cells) [8]. The experiments were performed in serum‐free conditions to mitigate the impact of endogenous TGFβ and other serum growth factors [25, 26, 27]. We found that hepsin specifically upregulates tEGFR levels in TGFβ1 expressing MCF10A cells (MCF10A‐pIND20‐HPN pL6‐TGFβ1V5, from here on MCF10A‐HT) (Fig. 3B). However, hepsin failed to induce tEGFR in the parental MCF10A cells not expressing TGFβ1 (Fig. S3B). Thus, the data suggest that hepsin requires TGFβ for upregulation of total‐EGFR.

To explore TGFβ1 downstream signaling mechanisms important for upregulation of tEGFR on a pathway triggered by hepsin, we included in the assays specific inhibitors for canonical and alternative TGF‐β signaling cascades. TGF‐β binds to specific receptors, TGFβR2 and TGFβR1 (ALK5), to initiate canonical Smad2/3 signaling. Alternatively, TGF‐β can bind to, for example, TGFβR2/ALK5—TGFβR2/ALK1/2 complexes to activate Smad1/5/8 signaling [28]. First, we observed that inhibition of ALK5 with a panel of inhibitors (Galunisertib/ALK5i, RepSox/ALK5i, A‐83‐01/ALK4/5/7i) completely abolished hepsin‐induced upregulation of tEGFR in MCF10A‐HT cells. However, we observed that ALK5 inhibition also upregulated tEGFR on its own in MCF10A cells (Fig. 3C and Fig. S3C), through a mechanism that was not studied further. Furthermore, we observed that also inhibition of ALK1/2/3/6 blocked hepsin‐mediated upregulation of tEGFR (Fig. 3D). The ALK1/2/3/6 inhibitor did not have any observable effects on the tEGFR levels on its own. The ALK1/2/3/6 inhibitor also suppressed phospho‐EGFR and phospho‐MAPK levels, indicating that these growth and proliferation pathways were activated by hepsin through TGFβ pathways (Fig. 3D). Both ALK1 protein and phospho‐Smad1/5 were expressed in the mammary epithelial MCF10A cells (Fig. S3D,E). We observed that constitutive TGFβ expression establishes lower p‐SMAD1/5 status than observed in the parental MCF10A cells. While this may be consistent with the idea of dynamic regulation of phospho‐Smad1/5 by TGFβ [29], the functional consequencs of this downmodulation for hepsin/TGB‐beta signaling remains to be studied.

Next, we determined the growth‐promoting roles of TGFβ and EGFR signaling downstream of hepsin by exploring the growth patterns of our genetically engineered MCF10A‐HT cells in 3D culture. Dox‐induced hepsin increased the growth of the MCF10A‐HT epithelial structures but not the parental cells devoid of TGFβ overexpression (Fig. 3E), suggesting that the growth‐promoting function of hepsin involves TGFβ. The dox‐induced hepsin failed to promote growth of the structures or increase mitosis marker phosphohistone 3 (pH3) in the presence of a hepsin function blocking antibody (Ab25), which corroborates the role of proteolytically active hepsin in mediating the enhanced proliferation (Fig. S3F,G). Treatment of TGFβ and hepsin overexpressing MCF10A structures with a small molecule ALK1/2/3/6 or EGFR inhibitor prevented their growth (Fig. 3E). In addition, an ALK1 function‐blocking antibody specifically prevented the hepsin‐dependent increase in the growth of MCF10A structures (Fig. 3F), suggesting a role for both that ALK1 and EGFR in the pro‐growth activity of hepsin.

Finally, we confirmed the role of EGFR signaling in the growth of MCF10A structures. A high EGFR inhibitor concentration completely abrogated the spheroid growth in the long‐term cultures (Fig. 3E). To more specifically address the role of hepsin‐dependent EGFR mediated growth signaling, we used a lower concentration (1 μm) and a shorter treatment period (24 h) for the EGFR inhibitor erlotinib. In short‐term assays with pH3 as a marker for mitotic activity, erlotinib specifically inhibited the hepsin‐dependent increase in the frequency of pH3 positive cells (Fig. 3G). Together, these findings are consistent with a functional importance of the TGFβ/ALK5/ALK1‐EGFR signaling axis in mediating the proliferation promoting effects of hepsin in mammary epithelial cells.

### 3.4 Hepsin inhibitors downregulate EGFR and TGFβ signaling in the breast cancer patient‐derived explant cultures (PDECs)

Next, we tested whether hepsin might regulate the TGFβ‐EGFR pathway in context of authentic human breast cancer tissue. For these experiments, we used patient‐derived explant culture (PDEC) system, which has been recently developed in our laboratory [16, 17, 18]. Briefly, PDECs are small, intact breast cancer fragments that preserve the tumor micro‐environment and can be cultured *ex vivo* for weeks in specific 3D matrices.

**Fig. 4:**
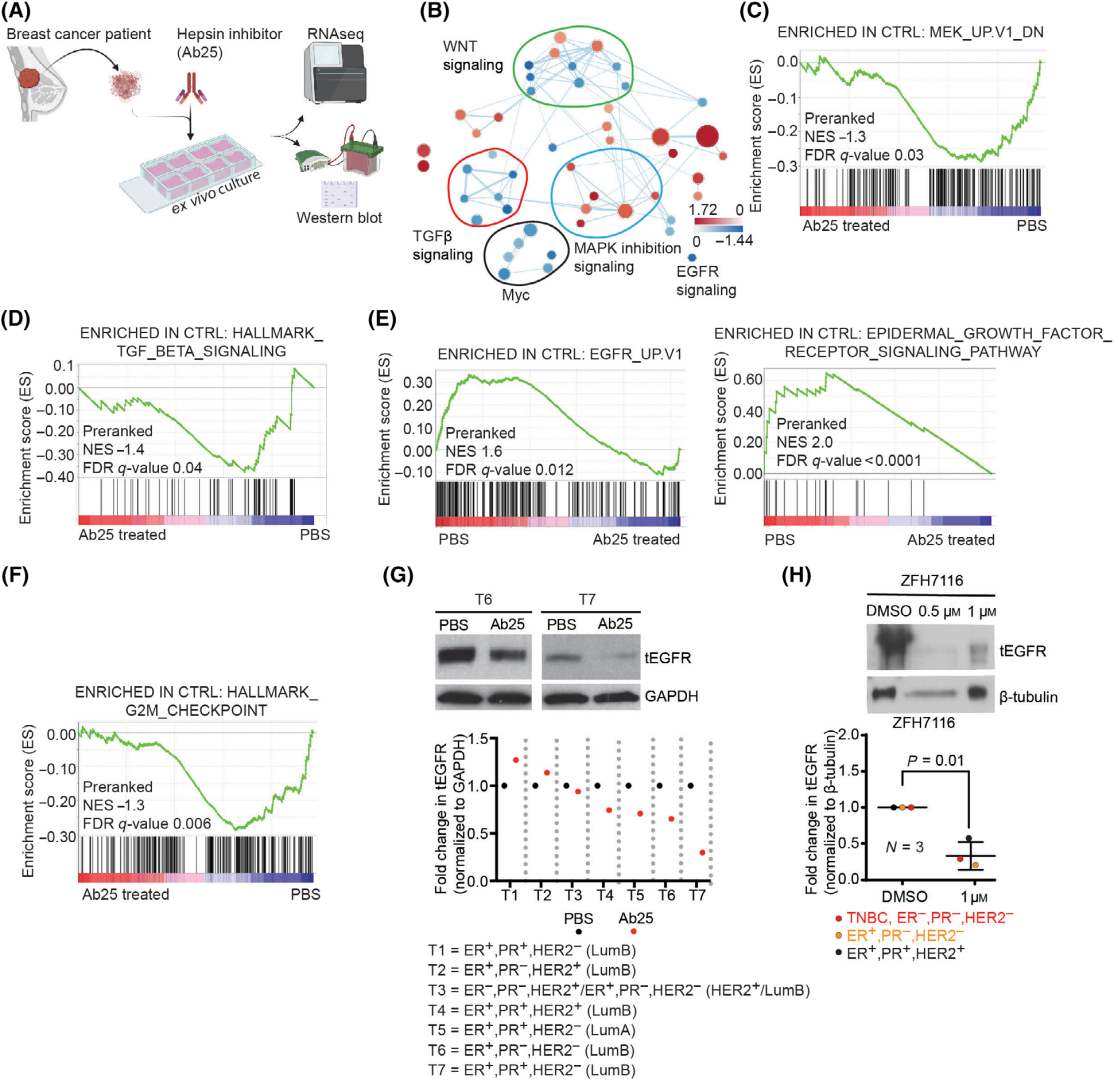
Hepsin targeting drugs inhibit TGFβ and EGFR signaling in human tumors. (A) Schematic depiction of the human patient‐derived explant culture (PDEC) treatment experiment with the hepsin inhibitor antibody Ab25. (B) Selected gene signatures of pathways that are up‐ or downregulated by Ab25 on mRNA level in PDECs. Each circle represents a gene signature. The blue color indicates the downregulation of the signature, and the red color indicates the upregulation. The size of nodes correlates with gene set size. (C–F) Gene set enrichment analysis (GSEA) plots show the downregulation of MEK, EGFR, TGFβ, and G2M pathways by the Ab25 treatment (NES‐ normalized enrichment score, FDRq‐ false discovery rate corrected significance). (G) Western blot analysis of total‐EGFR (tEGFR) levels in Ab25 treated PDECs compared to PBS (control) treated ones. The graph represents the quantitation of individual treatment experiments where T1 to T7 denoted PDECs from different patients (*N* = 7). The results are normalized to GAPDH and presented as fold change to PBS treated control. (H) Western blot analysis of tEGFR levels in hepsin small molecule inhibitor‐treated (ZFH7116) PDECs from three different patients. Significance was tested using the student's *t*‐test. Breast cancer subtypes are indicated in G and H. Data are presented as mean ± SD.

To test the role of hepsin in promotion of the TGFβ‐EGFR pathway in human cancer, we treated PDECs (Fig. S4A) derived from six different breast tumors with control (PBS) or with hepsin inhibitory antibody Ab25 for 48 h, after which RNA sequencing was performed (Fig. 4A). Gene Set Enrichment Analysis (GSEA) revealed that Ab25 treatment downregulates gene signatures corresponding to TGFβ and EGFR signaling (Fig. 4B and Fig. S4B). We also observed enrichment in signatures corresponding to MAPK pathway inhibition and proliferation (Fig. 4B and Fig. S4C,D), consistent with the data obtained in mouse mammary tumors (Fig. 1). The whole analysis is shown in Fig. S4C as a Cytoscape network representation, in which each node is divided into six sectors, each representing the score for PDECs from an individual patient. Figure 4C–F show signatures significantly downregulated by Ab25 (enriched in control). The results indicate that hepsin regulates TGFβ, MAPK, and EGFR signaling and cell proliferation in some but not necessarily all human breast tumors.

To further investigate and confirm the hepsin‐mediated regulation of EGFR in *ex vivo* human breast cancer cultures, we examined the EGFR protein levels in control and Ab25‐treated PDECs derived from an additional seven patients and found Ab25‐mediated EGFR downregulation in 5 out of 7 tumors (Fig. 4G). The role of hepsin in regulating EGFR protein levels was confirmed using a small‐molecule hepsin inhibitor ZFH7116 [11, 12], which downregulated EGFR levels in PDECs derived from primary breast tumors of three additional patients (Fig. 4H).

Altogether, the data shown in Fig. 4 indicate that hepsin promotes TGFβ and EGFR signaling, and upregulation of EGFR protein levels in authentic human breast tumor samples. These findings suggest hepsin as an important upstream activator of TGFβ and EGFR signaling and a possible new target for therapeutic strategies aiming to intervene these key pro‐tumorigenic signaling pathways in human cancer.

## 4 Discussion

The TGFβ pathway is a well‐established driver of such oncogenic phenomena as resistance to therapies [30] and immune evasion [31, 32, 33], making it an attractive target for breast cancer therapy. Proteolytic enzymes can directly activate latent‐TGFβ, but the evidence for this remains mostly limited to *in vitro* studies [34]. Furthermore, knockout mice for single proteases usually do not display any reduction in the TGFβ pathway activity [34]. The recent discovery of hepsin as a promoter of the TGFβ pathway in the normal mammary gland, and WAP‐Myc driven mammary tumors provides a promising new avenue for mammary‐specific pharmacological targeting of the TGFβ pathway [8].

The oncogenic role of hepsin in invasion is well established in the prostate [1, 9] and breast [3, 4] cancer, but the pathways responsible for hepsin‐driven growth [3, 35, 36] remain poorly understood.

The role of hepsin in promoting tumor growth and cell proliferation is unclear, since several conflicting reports on the effects of hepsin overexpression and inhibition on cell proliferation and tumor growth, ranging from hepsin being a suppressor of proliferation [37, 38, 39, 40] to promoter of proliferation [35, 36, 41]. Other studies report that hepsin overexpression drives invasion and metastasis without affecting tumor growth or cancer cell proliferation [1, 9]. Here we attempt to consolidate the contradictory reports based on the recently published link between hepsin and the TGFβ pathway.

Here, we present data suggeting that hepsin promotes primary and metastatic tumor growth and cell proliferation via the activation of the TGFβ and EGFR signaling pathways. However, the ability of hepsin to promote proliferation required simultaneous high expression level of TGFβ1 in the cells. The regulation of the TGFβ pathway by hepsin is interesting since this signaling pathway provides a possible explanation for the reported dual role of hepsin as a contextual suppressor or promoter of the cell proliferation and tumor invasion, namely the context depending on the other downstream signaling inputs received by the cells. This context‐dependency of TGFβ1 signaling is exemplarily illustrated by a study with Myc transformed fibroblasts, showing that the ability of TGFβ to promote proliferation crucially depends on platelet‐derived growth factor (PDGF) as PDGF alone has no effect, TGFβ alone acts as a suppressor, but the combination promotes proliferation [42]. Therefore, it is plausible that the outcome of the hepsin driven TGFβ signaling varies depending on the model and experimental conditions.

Our results are consistent with the previous findings that report anti‐proliferative effects of hepsin overexpression [37]. We show that indeed, overexpression of hepsin in MCF10A pIND20‐HPN cells reduced spheroid formation in 3D culture (Fig. 3E). However, when the overexpression of hepsin and TGFβ were combined, hepsin acted as a promoter of proliferation. This indicates that the ability of hepsin to promote proliferation is dependent on the context provided by the overexpression of TGFβ. Further investigation of the pathways revealed that hepsin activates EGFR and its downstream signaling. In fact, earlier studies have reported the upregulation of EGFR by TGFβ and the correlation between these two factors in human tumors, which have led to a speculative notion that these effects may explain some of the pro‐tumorigenic effects of the TGFβ pathway in breast cancer [22, 24].

## 5 Conclusion

Our data provide both functional and correlative evidence that the proliferative effects of hepsin are indeed dependent on the hepsin‐dependent regulation of the TGFβ pathway and that EGFR signaling is a critical downstream contributor to the oncogenic signaling by hepsin. We further demonstrate that hepsin inhibiting small molecule compounds and inhibitory antibodies may be used to intervene with these signaling pathways *in vitro* (Fig. S3) and in the patient‐derived explant cultures, which offers translationally relevant leads to follow towards successful targeting of oncogenic serine proteases.

## Conflict of interest

The authors declare no conflict of interest.

## Author contributions

DB designed the study, performed the experiments, provided resources, curated data, validated, investigated, contributed to methodology and wrote the manuscript; PM designed the study, performed the experiments, provided resources, curated data, validate, investigated, contributed to methodology and wrote parts of the manuscript; SMP provided resources, curated data, validated, investigated, contributed to methodology, and wrote the original draft; JMA performed the experiments, provided resources, curated data, validated, investigated, contributed to methodology and wrote the original draft; IS curated the data, was involved in formal analysis, performed histological evaluation of tissues, contributed to methodology, and wrote the original draft. KB curated the data, was involved in formal analysis, performed histological evaluation of tissues, contributed to methodology, and wrote the original draft., DK and JJ provided resources, contributed to methodology and wrote the original draft; TK curated the data, was involved in data analysis and writing of the original manuscript; SJ validation of hypotheses, contributed to methodology, wrote the original draft. JP designed the study, curated data, and wrote the original draft; TT designed the study, performed the experiments, provided resources, curated data, validated, investigated, contributed to methodology, and wrote the manuscript; JK designed the study, provided resources, curated data, validated, investigated, contributed to methodology, and wrote the manuscript. All authors carefully reviewed the manuscript and approved the final version.

### Peer review

The peer review history for this article is available at https://www.webofscience.com/api/gateway/wos/peer‐review/10.1002/1878‐0261.13545.

## Supporting information


**Fig. S1.** Growth of primary and metastatic tumors in mice syngrafted with WT or *Hpn* KO WAP‐ Myc tumors.
**Fig. S2.** Gene set enrichment analysis (GSEA) of WT and *Hpn* KO mammary tumors.
**Fig. S3.** Total EGFR protein levels in WAP‐Myc tumor cells, and total EGFR, phospho‐SMAD1/5 and ALK1 protein levels in MCF10A‐based cell lines and the effects of Galunisertib (ALK5 inhibitor) and Ab25, a hepsin neutralizing antibody.
**Fig. S4.** Histological features of patient derived explant cultures (PDEC)s and Cytoscape analysis of Gene Set Enrichment Analysis (GSEA) results.

## Data Availability

The data that support the findings of this study are available in Figs S1–S4. The RNAseq data that support the findings of this study are openly available in NCBI's Gene Expression Omnibus and are accessible through https://www.ncbi.nlm.nih.gov/geo/query/acc.cgi?acc=GSE164510, GEO Series accession number GSE164510, and https://www.ncbi.nlm.nih.gov/geo/query/acc.cgi?acc=GSE205774, GEO Series accession number GSE205774, and https://www.ncbi.nlm.nih.gov/geo/query/acc.cgi?acc=GSE245265, GEO Series accession number GSE245265.
