# Supplementary material for: Hepsin promotes breast tumor growth signaling via the TGFβ‐EGFR axis
Source: Mol Oncol. 2023 Nov 13;18(3):547–61. doi: 10.1002/1878-0261.13545 (PMC10920082; doi:10.1002/1878-0261.13545)
Supplement: Supplementary file 1 — Fig. S1. Growth of primary and metastatic tumors in mice syngrafted with WT or Hpn KO WAP‐ Myc tumors. Fig. S2. Gene set enrichment analysis (GSEA) of WT and Hpn KO mammary tumors. Fig. S3. Total EGFR protein levels in WAP‐Myc tumor cells, and total EGFR, phospho‐SMAD1/5 and ALK1 protein levels in MCF10A‐based cell lines and the effects of Galunisertib (ALK5 inhibitor) and Ab25, a hepsin neutralizing antibody. Fig. S4. Histological features of patient derived explant cultures (PDEC)s and Cytoscape analysis of Gene Set Enrichment Analysis (GSEA) results. [file MOL2-18-547-s001.docx]

**SUPPLEMENTARY FIGURES AND FIGURE LEGENDS**


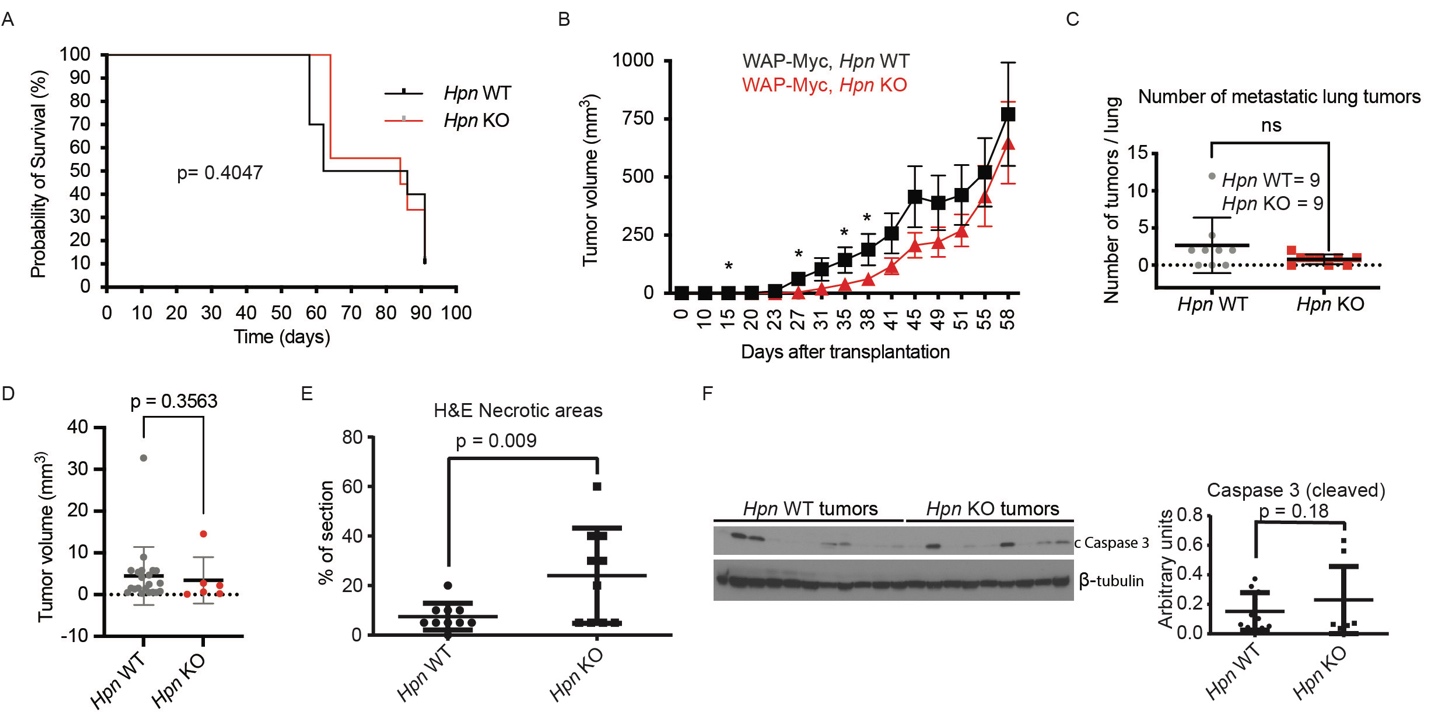


**Supplementary Figure 1. Growth of primary and metastatic tumors in mice syngrafted with WT or *Hpn* KO WAP- Myc tumors**. (A) Kaplan-Meier plot of the WT and *Hpn* KO WAP-Myc tumor bearing mice. P-value was calculated with Gehan-Breslow-Wilcoxon test. (B) Growth curves of *Hpn* WT (N = 16) and *Hpn* KO (N = 14) WAP-Myc tumors in *Hpn* WT recipient mice. Tumor volumes are presented as averages (mean±SEM) per group, the timeline specifying days after the transplantation. Statistical significance from student's t-test, * denotes p<0.05. (C) The graph shows the number of metastatic lung tumors per lung in WT mice harboring either WT or *Hpn* KO tumors. One dot represents the number of metastatic lung nodules in one mouse (mean ±SD, N=9 for *Hpn* WT and N=9 for *Hpn* KO). (D) The graph shows the raw data for metastatic lung tumors with two outliers (mean ±SEM, N=21 for *Hpn* WT and N=6 for *Hpn* KO, p-value determined with Student’s t-test). The data after exclusion of the two outliers (Iglewicz and Hoaglin's robust test for multiple outliers) are shown in Figure 1C. (E) The graph shows the proportion of H&E-stained necrotic areas in formalin fixed tumor sections (mean ±SD, N=9 for *Hpn* WT and N=10 for *Hpn* KO, p-value was determined with Student’s t-test). (F) Immunoblot analysis for cleaved (c) Caspase 3 with β-tubulin as a loading control. The graph on the right shows loading normalized quantitation of the immunoblot on the left (mean ±SEM, N=11 for *Hpn* WT and N=9 for *Hpn* KO, p-value was determined with Student’s t-test).


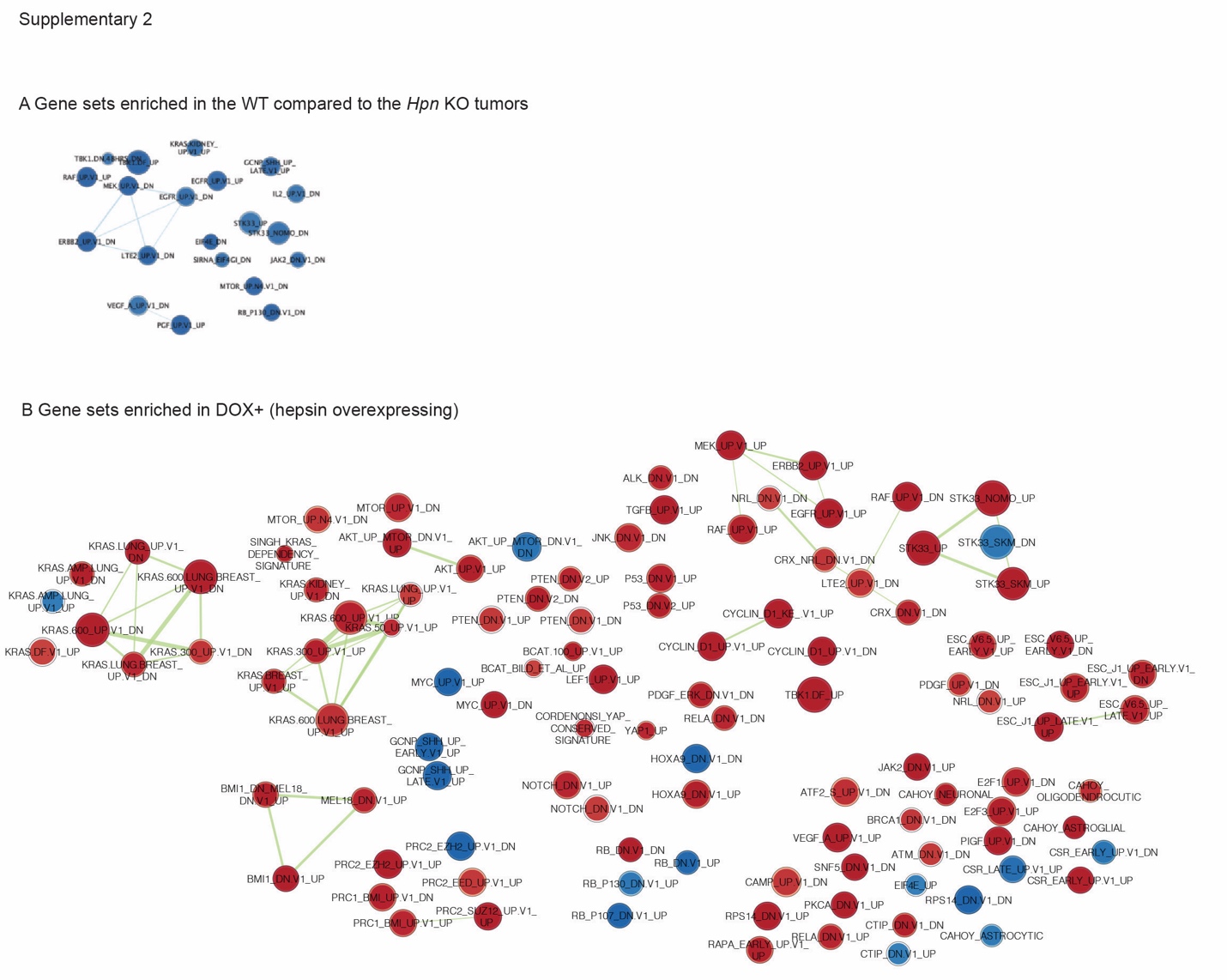


**Supplementary Figure 2. Gene set enrichment analysis (GSEA) of WT and *Hpn* KO mammary tumors**. (A) Selected gene signatures downregulated by loss of hepsin in WAP-Myc driven tumors from Figure 2A. The size of the nodes indicates the size of signatures. The blue color indicates the downregulation induced by hepsin knockout. (B) GSEA of public data (8); GEO dataset GSE164510), comparing mammary tumors overexpressing hepsin (DOX+) to control tumors (DOX-) from Figure 2H.


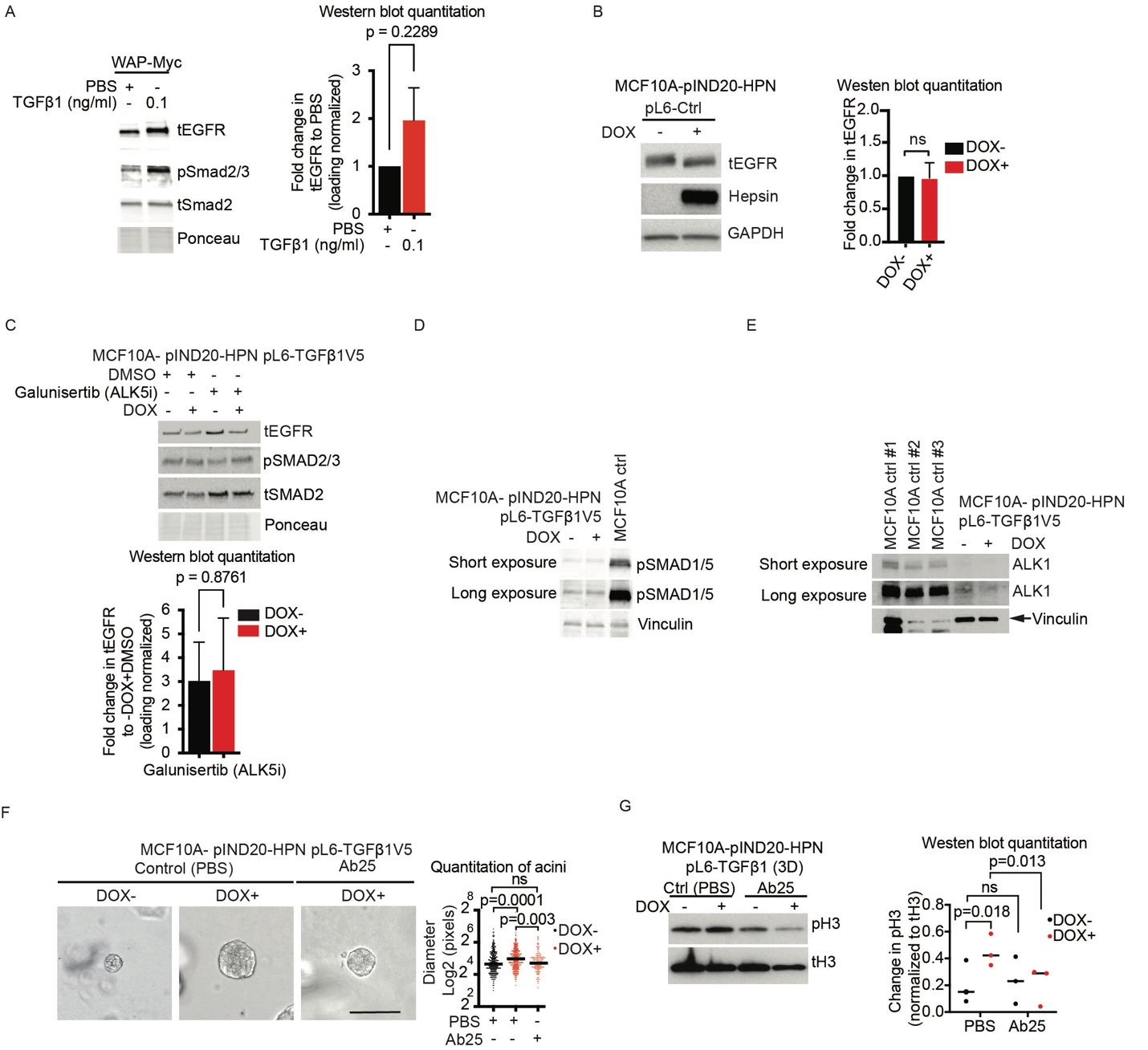


**Supplementary Figure 3. Total EGFR protein levels in WAP-Myc tumor cells, and total EGFR, phospho-SMAD1/5 and ALK1 protein levels in MCF10A-based cell lines and the effects of Galunisertib (ALK5 inhibitor) and Ab25, a hepsin neutralizing antibody**. (A) Representative western blot analysis of total-EGFR (tEGFR), phospho-SMAD2/3 (pSMAD2/3), and total-SMAD2 (tSMAD2) in WAP-Myc tumor cells. The cells were treated with 0.1 ng/mL recombinant TGFβ1 for 6 hours with PBS treatment as a control. The histogram shows quantitation of tEGFR levels from western blots, representing 3 biological replicates (bands normalized to loading). Shown is mean±SEM, p-value was derived from unpaired t-test). (B) Representative western blot analysis of tEGFR in control (DOX-; N=4) and hepsin overexpressing (DOX+; N=4) MCF10A-pIND20-HPN cells. Data are presented as mean ± SD. Significance tested with the student's t-test. (C) Representative western blot analysis of tEGFR in MCF10A-pIND20-HPN pL6-TGFβ1V5 cells. The cells were treated with ALK5 inhibitor (i) (10 μM Galunisertib) for 48 h with (DOX+; 1 μg/mL) or without (DOX-, control) hepsin overexpression. The histogram data are presented as mean ±SD. Significance was tested using unpaired t-test (N=4). (D) Western blot analysis of phospho-SMAD1/5 (pSMAD1/5) in MCF10A-pIND20-HPN pL6-TGFβ1V5 cells with (DOX+; 1 μg/mlL) or without (DOX-, control) hepsin overexpression and parental MCF10A cells. Vinculin was used as a loading control. The data are representative of three biological replicates. (E) Western blot analysis of ALK1 in MCF10A-pIND20-HPN pL6-TGFβ1V5 cells with (DOX+; 1 μg/mL) or without (DOX-, control) hepsin overexpression and three biological replicates of parental MCF10A cells (ctrl #1 to #3). Vinculin was used as a loading control. ns, not significant. (F) Phase contrast microscopy images of MCF10A-pIND20-HPN pL6- TGFβ1V5 cells cultured in 3D Cultrex for two weeks with (DOX+) or without (DOX-) hepsin overexpression. 5 μM hepsin inhibitory antibody Ab25 was used as indicated in the figure. Experiments were repeated three times, with at least 100 epithelial structures counted per group in each repeat (one dot represents one structure). The black line denotes the mean. Significance was tested using the student's t-test. Scale bar is 100 μm. (G) Western blot analysis of phospho-H3 (pH3) and total H3 (tH3) in lysates prepared from 3D cultured MCF10A-pIND20-HPN pL6- TGFβ1V5 with (DOX+) and without (DOX-) hepsin overexpression either in the presence of Ab25 or PBS control. The black line denotes the mean. One dot represents one independent experiment. Significance was tested with unpaired t-test.


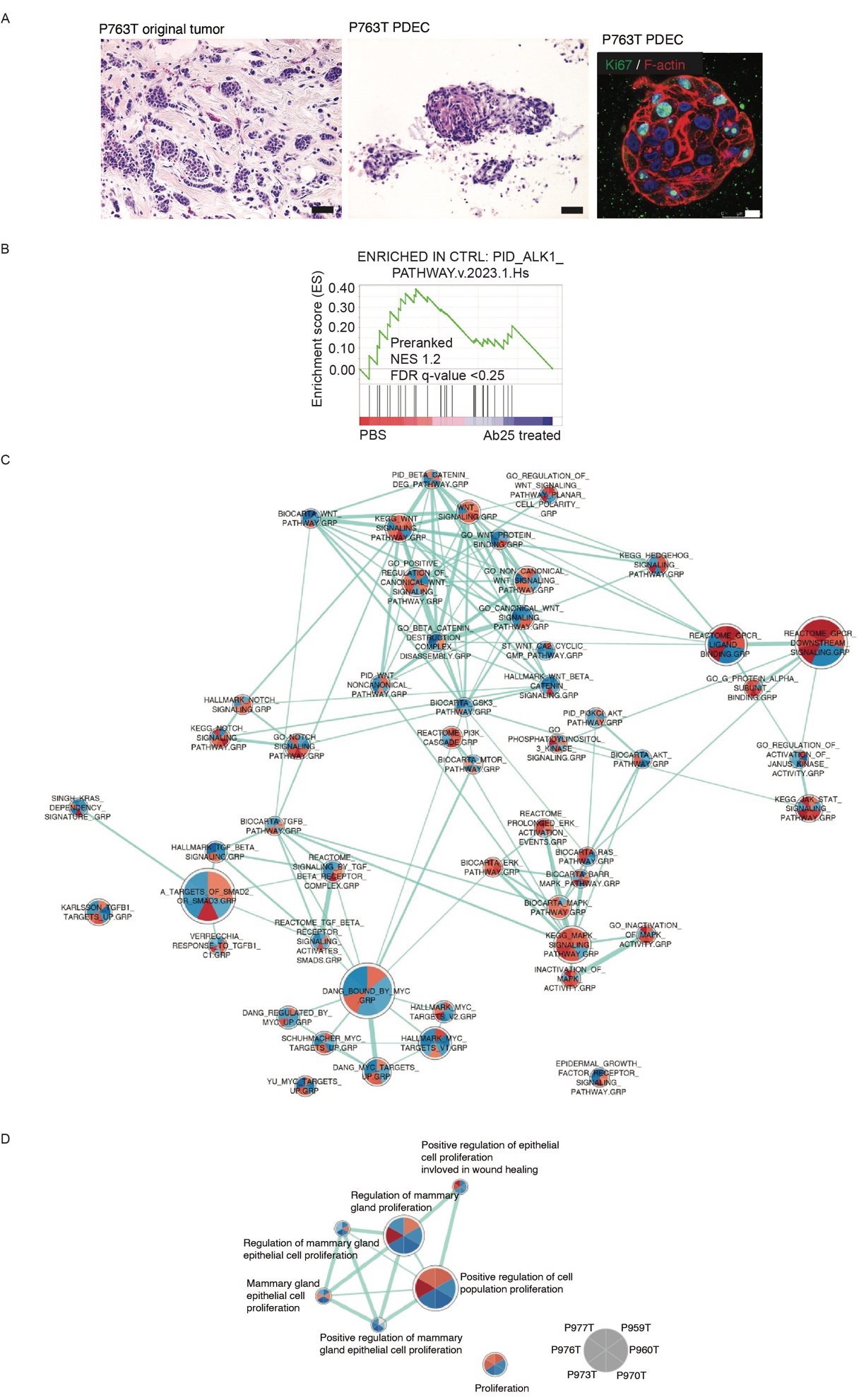


**Supplementary Figure 4. Histological features of patient derived explant cultures (PDEC)s and Cytoscape analysis of Gene Set Enrichment Analysis (GSEA) results.** (A) H&E-stained histological sections of the original tumor and the corresponding PDEC-BC sample (H&E staining left and middle panel; right: IF for Ki67 and f-actin), which was cultured in a 3D culture for 7 days. Scale bar (IHC figures)= 50 μm, Scale bar (IF figure)= 10 μm. (B) Gene set enrichment analysis (GSEA) plot shows the downregulation of ALK1 pathway by the Ab25 treatment (NES- normalized enrichment score, FDRq- false discovery rate corrected significance). (C and D) Cytoscape-based GSEA analysis derived from RNAseq data collected after PDEC treatment with Ab25 hepsin inhibitory antibody. Each circle represents a gene signature and is divided into six sectors. Each sector represents the effect of the Ab25 antibody treatment on one tumor. (C) The gene sets that are significantly downregulated after Ab25 treatment (FDR q = <0.25). (D) The gene sets related to cell proliferation that are significantly downregulated after Ab25 treatment (FDR q = <0.25). For D**,** also the patient map is provided (a grey pie chart with different sectors corresponding to different patients). In C and D, the blue color of the sector indicates downregulation of this signature in the tumor, and the red color indicates upregulation.
